# Changes in risk factors for food sensitization in early life: Analysis over a period of 10 years

**DOI:** 10.3389/fimmu.2023.1153607

**Published:** 2023-03-31

**Authors:** Zhuoying Ma, Lin Chen, Ruoling Xian, Heping Fang, Jing Chen, Haiqi Li, Juan Wang, Yan Hu

**Affiliations:** ^1^ Department of Child Health Care, Children’s Hospital of Chongqing Medical University, Chongqing, China; ^2^ National Clinical Research Center for Child Health and Disorders, Ministry of Education Key Laboratory of Child Development and Disorders, Chongqing Key Laboratory of Child Health and Nutrition, Chongqing, China

**Keywords:** children, food sensitization, risk factor, change, early life

## Abstract

**Background:**

Although epidemiological trends of childhood food sensitization (FS) in IgE-mediated food allergy were reported in China, few studies have examined at changes in its risk factors.

**Objective:**

To investigate the change in early-life risk factors associated with childhood food sensitization during 2009–2019 in China.

**Methods:**

Data from two cross-sectional surveys conducted in 2009 and 2019 (401 and 513 children, respectively) were analyzed. The results of skin prick tests and information on food sensitization-related risk factors in children were summarized, including family history of atopic disease (FHA), demographic characteristics, method of delivery, feeding patterns, sibship size, pet ownership, and vitamin D supplementation. Binary logistic regression was used to calculate the odds ratio and the regression coefficient *β*-value of risk factors in the 2009 and 2019 surveys separately. Then, coefficient *β*-value differences between the two surveys were analyzed by the bdiff command in STATA to describe the change in risk factors over 10 years.

**Results:**

The 2009 survey revealed that FHA, age, only child, and feeding patterns were associated with food sensitization. The 2019 survey showed that food sensitization was affected by age, sex, and feeding patterns. However, from 2009 to 2019, the probability of food sensitization in the only-child group significantly increased by 226.0% (*β*-value difference = 0.81, *P* = 0.024) and decreased by 65.0% in female children (*β*-value difference = −1.06, *P* = 0.008). The effect of age on food sensitization decreased by 50.0% (*β*-value difference = −0.69, *P* < 0.001) over 10 years.

**Conclusion:**

The effect of FHA and common lifestyle factors on food sensitization did not significantly change during 2009−2019. However, the influence of demographic characteristics on food sensitization has changed since 2009; that is, older age, male gender, and only child are more likely to develop food sensitization, which needs to be considered in future epidemiological surveys.

**Clinical Trial Registration:**

http://www.chictr.org.cn/, identifier ChiCTR1900024338.

## Introduction

Food allergy is part of the second wave of atopic diseases after asthma and allergic rhinitis (1). The prevalence of IgE-mediated food allergy has been increasing, particularly in infants and young children, affecting up to 10% of individuals in industrialized countries and 7.6% in China (2, 3). The current treatment mainly involves “passive” avoidance of specific food proteins or “active” repeated induction of food antigen stimulation to achieve oral immune tolerance. However, the elimination of food allergens from the diet can cause nutrition-related problems. Furthermore, active treatment, including oral immunological therapy, needs to be provided in a rigorously monitored medical setting, limiting its clinical application in resource-poor settings. Therefore, understanding the early-life risk factors for food allergies is crucial for their prevention and management.

In China, a rapid increase in the prevalence of IgE-mediated food allergy in children has been reported since the 1990s, which gradually stabilized after 2010 (3). The influence of genetic factors on the shift in the epidemiological trend of food allergy has not yet been elucidated. Despite the complex food allergy pathogenesis, immunological tests have provided a wealth of information on IgE-mediated food allergy. Food sensitization, which is a condition characterized by a positive result for specific IgE-mediated tests (skin prick test or allergen-specific IgE) without clinical symptoms after food allergen ingestion, is involved in the development of IgE-mediated food allergy. The food sensitization prevalence trend is consistent with that of food allergy in many countries, including the United States, Australia, the Netherlands, and China (3–6). Therefore, food sensitization and food allergy may share similar risk factors. We hypothesize that as the prevalence of food sensitization and food allergy changes over time, the factors influencing them also change.

Risk factors in early life that impact the development of childhood atopic diseases have been reported in different literature, including family history of atopic history (FHA), sex, maternal pregnancy status, mode of delivery, infant diet, and pet ownership (7–12). However, data on the changes in the risk factors for food sensitization are lacking.

In this study, epidemiological data on food sensitization obtained from surveys conducted in 2009 and 2019 were used to investigate the changes in risk factors influencing food sensitization over a period of 10 years. To the best of our knowledge, this is the first comparative study that describes the changes in the risk factors for food sensitization based on data from studies done in different years. It will provide a basis for further research into the development of strategies for preventing food allergies.

## Materials and methods

### Participants and study design

Data from children aged 0-24 months who participated in the 2009 and 2019 food allergy epidemiological surveys were collected for this paper. They were enrolled during well-baby check-ups at the division of Child Health Care of Children’s Hospital affiliated to Chongqing Medical University, and the skin prick test (SPT) was performed on all eligible participants. Their parents/guardians provided informed consent, and the studies were carried out in accordance with the principles of the Declaration of Helsinki. In accordance with the national basic public health policy, children under the age of 2 have at least seven routine medical examinations (13). The Children’s Hospital of Chongqing Medical University is the largest children’s hospital in the Sichuan-Chongqing region, and the sources of subjects were consistent with the community population in the two surveys. More details are described in the previously published article (3).

**Figure 1:**
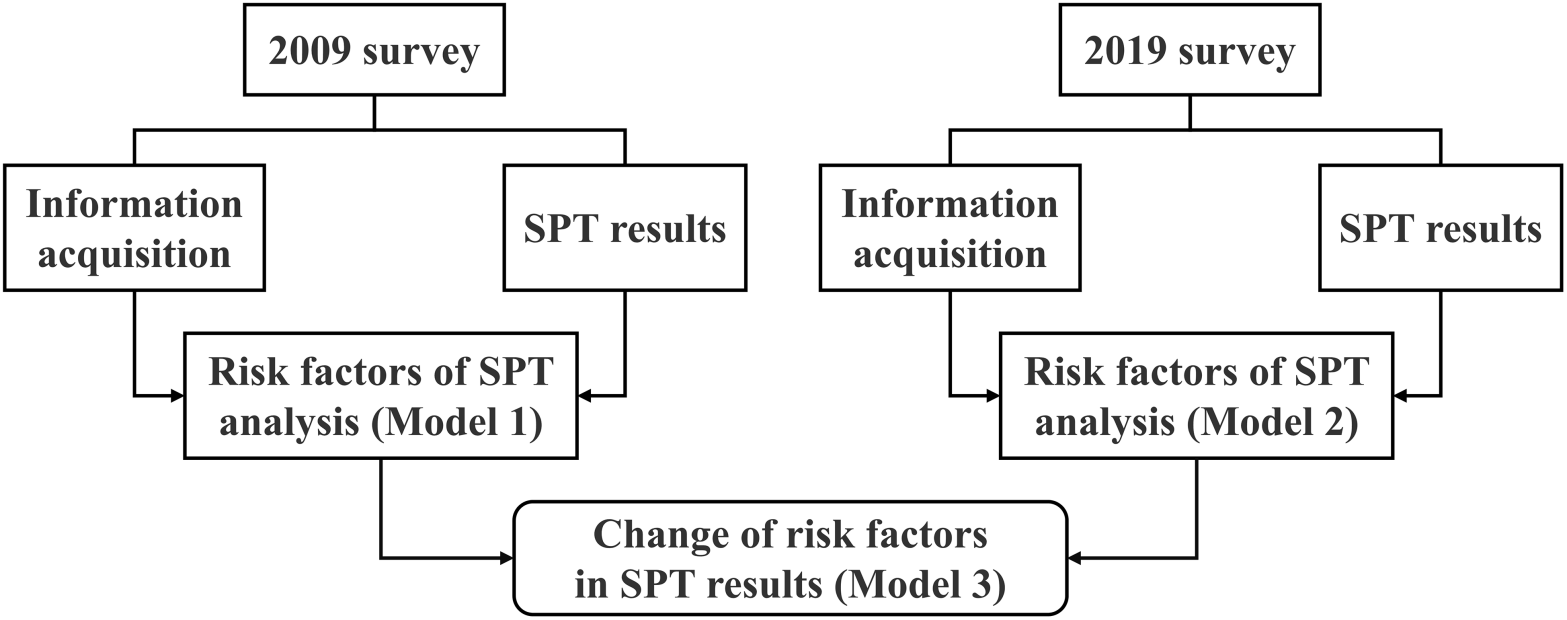
Study design and statistical process comparing change in food sensitization risk factors between the 2009 and 2019 surveys. SPT, Skin prick test.

In this paper, demographic characteristics, lifestyle factors, and SPT results of the epidemiological survey in 2009 and 2019 were first summarized. Then, factors affecting SPT results in the two surveys were analyzed respectively to compare the differences. Finally, the differences in regression coefficient *β*-values between the two surveys were analyzed by the STATA command bdiff to determine whether the factors affecting SPT results changed during the 10 years. The process followed in this study is shown in **Figure 1**.

### Information acquisition

In both surveys, investigators collected related information about the children using paper-based questionnaires, including data on demographic factors (sex, age, number of siblings, gestational age, mode of delivery, and birth weight), FHA, infant diet (exclusive breastfeeding or non-exclusive breastfeeding in two different periods, time of solid food introduction, and vitamin D supplementation), prenatal environment (smoke exposure and diet during pregnancy), and pet ownership.

### Food sensitization assessment

Food sensitization was defined as a positive SPT result to any food allergens, where the average diameter of the wheal was greater than that of the negative control (≥3 mm). In the 2009 and 2019 surveys, SPT antigen extracts included egg yolk, egg white, milk, soybean, peanut, wheat, fish, shrimp, orange, and carrot (purchased from the American Greer Company). The positive and negative controls were set to 10 mg/ml of histamine phosphate and normal saline, respectively.

### Statistical methods

Power analysis for sample size and logistic regression coefficient β-values was calculated using the PASS 15.0 software, and the results showed sufficient statistical power (>0.85) to support the findings.

STATA 15.0 software was used for the statistical analyses. First, for the data of 2009 and 2019, binary logistic regression models 1 and 2 were established to analyze the risk factors affecting SPT results in the two surveys, respectively. Then the regression coefficient β-values and odds ratio (OR) of models 1 and 2 were obtained. Further analysis (model 3) was applied to compare the differences in regression coefficient β-values between the models (STATA command: bdiff) to investigate whether significant changes had occurred in the probability of risk factors influencing the SPT results over 10 years (14). A *P*-value less than 0.05 was considered to indicate statistically significant differences or results.

### Ethics approval

The study was approved by the Medical Research Ethics Committee of Children’s Hospital affiliated to Chongqing Medical University.

## Results

### Survey characteristics

In the 2009 survey, a total of 401 children were recruited: 317 were 0–12 months old and 84 were 13–24 months old. In the 2019 survey, a total of 513 infants were recruited: 379 were 0–12 months old and 134 were 13–24 months old. **Table 1** shows the demographic characteristics and lifestyle factors of the participants from the two surveys. Although the data were from two cross-sectional surveys of food allergy in the same region and age group, it can be seen that there were significant differences in demographic characteristics and lifestyle factors between children participating in the 2009 and 2019 surveys, except for age, gender, FHA, and pet ownership. However, in the previous article, we reported that the incidence of food sensitization showed no change from 2009 to 2019 (18.0% *vs*. 15.6%; *χ* ^2^ = 0.905, *P* = 0.342) (3). It is suggested that the change in lifestyle factors may not be enough to affect the occurrence of food sensitization in the past 10 years.

**Table 1:** Distribution of risk factors in children between the 2009 and 2019 surveys.

Variable		2009 survey (*N* = 401), *n* (%)	2019 survey (*N* = 513), *n* (%)	*P*
Age (months)	Mean ± SD	8.14 ± 0.28	9.48 ± 0.25	0.750
Birth weight (kg)	Mean ± SD	3.31 ± 0.02	3.21 ± 0.02	0.020
Gender	Female	183 (45.64)	245 (47.76)	0.523
	Male	218 (54.36)	268 (52.24)	
FHA^a^	Positive	116 (28.93)	172 (33.53)	0.137
	Negative	285 (71.07)	341 (66.47)	
Only child	Yes	44 (10.97)	166 (32.36)	<0.001
	No	357 (89.03)	347 (67.64)	
Gestation age	Full-term	381 (95.01)	467 (91.03)	0.021
	Preterm	20 (4.99)	46 (8.97)	
Cesarean section	Yes	320 (79.80)	288 (56.14)	<0.001
	No	81 (20.20)	225 (43.86)	
Parental smoking	Yes	61 (15.21)	43 (8.38)	0.001
	No	340 (84.79)	470 (91.62)	
Pet ownership	Yes	33 (8.23)	32 (6.24)	0.245
	No	368 (91.77)	481 (93.76)	
High-protein diet during pregnancy^b^	Yes	152 (37.91)	246 (47.95)	0.002
	No	249 (62.09)	267 (52.05)	
Non-exclusive BF in the first 3 days	Yes	271 (67.58)	227 (44.25)	<0.001
	No	130 (32.42)	286 (55.75)	
Non-exclusive BF for at least 4 months	Yes	233 (58.10)	203 (39.57)	<0.001
	No	168 (41.90)	310 (60.43)	
Vitamin D supplementation	Yes	136 (33.92)	391 (76.22)	<0.001
	No	265 (66.08)	122 (23.78)	
Age of solid food introduction	Never^c^	113 (28.18)	152 (29.63)	<0.001
	0-4 months	34 (8.48)	10 (1.95)	
	4-6 months	196 (48.88)	121 (23.59)	
	≧6 months	58 (14.46)	230 (44.83)	

The differences in demographic characteristics and lifestyle factors between the two surveys did not affect the objective of exploring the change in food sensitization factors over a 10-year period.

FHA, Family history of atopic disease; BF, Breastfeeding.

a FHA includes asthma, atopic dermatitis, allergic rhinitis, and food allergies of their parents and siblings.

b Such as more than 3 eggs or 1 L of milk daily.

c For various reasons (e.g., young age or solid food refusal), the child had not been introduced to solid foods when the study was conducted.

Next, a chi-square analysis was conducted to further clarify the influencing factors of food sensitization between the two surveys (**Table 2**). The result showed that the factors, such as gender, only child, vitamin D supplementation, and age of solid food introduction, were different in children with food sensitization between 2009 and 2019.

**Table 2:** Distribution of risk factors in children with food sensitization between the 2009 and 2019 surveys.

Variable		2009 survey (*N* = 72), *n* (%)	2019 survey (*N* = 80), *n* (%)	*P*
Age (months)	Mean ± SD	11.85 ± 4.65	10.36 ± 5.82	0.455
Birth weight (kg)	Mean ± SD	3.36 ± 0.39	3.32 ± 0.46	0.342
Gender	Female	36 (50.00)	29 (36.25)	0.087
	Male	36 (50.00)	51 (63.75)	
FHA	Positive	30 (41.67)	30 (37.50)	0.600
	Negative	42 (58.33)	50 (62.50)	
Only child	Yes	65 (90.28)	25 (31.25)	<0.001
	No	7 (9.72)	55 (68.75)	
Gestation age	Full-term	71 (98.61)	77 (96.25)	0.364
	Preterm	1 (1.39)	3 (3.75)	
Cesarean section	Yes	60 (83.33)	45 (56.25)	<0.001
	No	12 (16.67)	35 (43.75)	
Parental smoking	Yes	12 (16.67)	6 (7.50)	0.081
	No	60 (83.33)	74 (92.50)	
Pet ownership	Yes	4 (5.56)	5 (6.25)	0.856
	No	68 (94.44)	75 (93.75)	
High-protein diet during pregnancy	Yes	34 (47.22)	38 (47.50)	0.973
	No	38 (52.78)	42 (52.50)	
Non-exclusive BF in the first 3 days	Yes	41 (56.94)	37 (46.25)	0.188
	No	31 (43.06)	43 (53.75)	
Non-exclusive BF for at least 4 months	Yes	28 (38.89)	23 (28.75)	0.186
	No	44 (61.11)	57 (71.25)	
Vitamin D supplementation	Yes	15 (20.83)	61 (76.25)	<0.001
	No	57 (79.17)	19 (23.75)	
Age of solid food introduction	Never	3 (4.17)	10 (12.50)	<0.001
	0-4 months	4 (5.55)	1 (1.25)	
	4-6 months	48 (66.67)	25 (31.25)	
	≧6 months	17 (23.61)	44 (55.00)	

### Risk factors of food sensitization over 10 years

**Figure 2:**
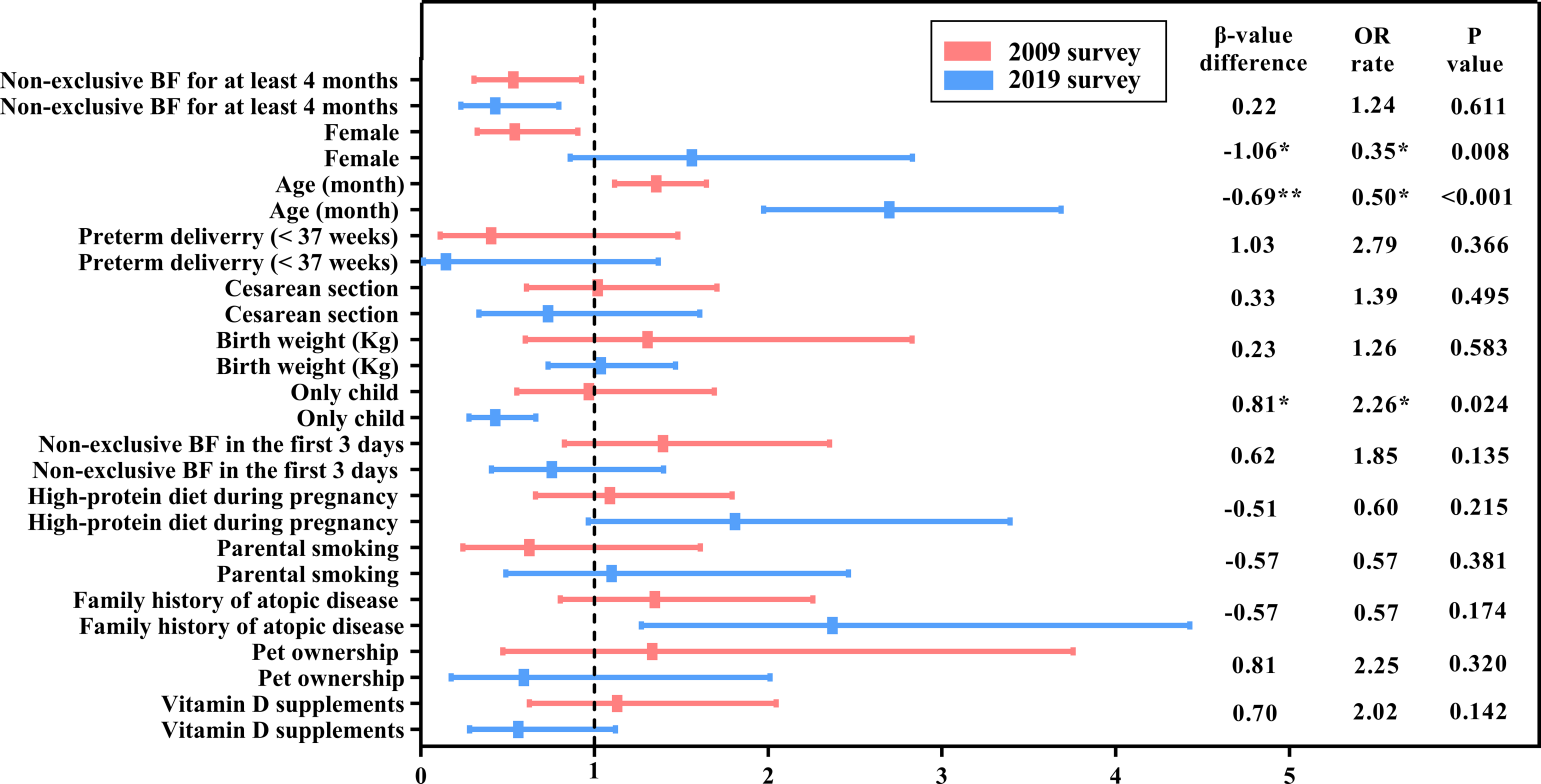
A comparison of the change in food sensitization risk factors between the 2009 and 2019 surveys. BF, Breastfeeding; OR, Odds ratio. The names of the variables in the table (including the results of the classification) are calculated in the model, except for age, which is classified as a rank variable from younger to older. P-value * means P<0.05; ** means P<0.01.

Binary logistic regression was used to understand the influencing factors of SPT results in 2009 and 2019, respectively. The results are shown in **Table 3** (columns 2-5) and **Figure 2**.

**Table 3:** A comparison of the change in food sensitization risk factors between the 2009 and 2019 surveys.

Variables	2009 survey		2019 survey		OR rate (2009/2019)	Change in factors	
	*β*-Value (95% CI)	OR (95% CI)	*β*-Value (95% CI)	OR (95% CI)		*β*-Value difference	*P*
Female	0.45 (−0.15 to 1.04)	1.56 (0.86 to 2.83)	−0.61 (−1.13 to −0.10)	0.54 (0.33 to 0.90)*	0.35*	−1.06*	0.008
Age (months)	0.99 (0.68 to 1.31)	2.70 (1.97 to 3.69)*	0.30 (0.11 to 0.50)	1.36 (1.12 to 1.65)*	0.50*	−0.69**	<0.001
Preterm delivery (<37 weeks)	−1.93 (−4.17 to 0.31)	0.15 (0.02 to 1.37)	−0.90 (−2.20 to 0.39)	0.41 (0.11 to 1.48)	2.79	1.03	0.366
Birth weight (kg)	0.04 (−0.31 to 0.38)	1.04 (0.73 to 1.47)	0.27 (−0.51 to 1.04)	1.31 (0.60 to 2.83)	1.26	0.23	0.583
FHA	0.86 (0.24 to 1.49)	2.37 (1.27 to 4.43)*	0.30 (−0.22 to 0.82)	1.35 (0.80 to 2.26)	0.57	−0.57	0.174
Cesarean section	−0.31 (−1.09 to 0.47)	0.73 (0.34 to 1.61)	0.02 (−0.50 to 0.53)	1.02 (0.61 to 1.71)	1.39	0.33	0.495
Parental smoking	0.09 (−0.71 to 0.90)	1.10 (0.49 to 2.46)	−0.47 (−1.42 to 0.48)	0.63 (0.24 to 1.61)	0.57	−0.57	0.381
Only child	−0.85 (−1.28 to −0.41)	0.43 (0.28 to 0.66)*	−0.03 (−0.59 to 0.53)	0.97 (0.55 to 1.69)	2.26*	0.81*	0.024
Pet ownership	−0.52 (−1.74 to 0.70)	0.59 (0.18 to 2.01)	0.29 (−0.75 to 1.32)	1.33 (0.47 to 3.76)	2.25	0.81	0.320
High-protein diet during pregnancy	0.59 (−0.04 to 1.22)	1.81 (0.96 to 3.39)	0.09 (−0.41 to 0.58)	1.09 (0.66 to 1.79)	0.60	−0.51	0.215
Non-exclusive BF in the first 3 days	−0.28 (−0.90 to 0.34)	0.75 (0.41 to 1.40)	0.33 (−0.19 to 0.86)	1.40 (0.83 to 2.35)	1.85	0.62	0.135
Non-exclusive BF for at least 4 months	−0.85 (−1.46 to −0.23)	0.43 (0.23 to 0.79)*	−0.63 (−1.18 to −0.08)	0.53 (0.31 to 0.93)*	1.24	0.22	0.611
Vitamin D supplementation	−0.58 (−1.27 to 0.11)	0.56 (0.28 to 1.12)	0.12 (−0.47 to 0.72)	1.13 (0.63 to 2.05)	2.02	0.70	0.142

The names of the variables in the table (including the classification results) are calculated in the model, except for age, which is classified as a rank variable from younger to older.

FHA, family history of atopic disease; BF, breastfeeding; OR, odds ratio; CI, confidence interval.

* means P < 0.05; ** means P < 0.001.

In 2009, the risk of food sensitization increased 2.7 times with age [OR = 2.70, 95% confidence interval (CI): 1.97–3.69], and it was 2.37 times higher in children with FHA than in those without FHA (OR = 2.37, 95% CI: 1.28–4.43). However, the risk of food sensitization was low in the only-child and non-exclusive breastfeeding infants for at least 4 months of age (OR = 0.43 for both). Although food sensitization was more common in female children, there was no statistical significance between male and female children (OR = 1.56, 95% CI: 0.86−2.83, *P* > 0.05).

In the 2019 survey, the factors influencing the SPT results were different from those in 2009. Age was still a risk factor for food sensitization, but the OR value decreased from 2.70 in 2009 to 1.36 in 2019. Non-exclusive breastfeeding for at least 4 months still affected SPT results (OR = 0.53, 95% CI: 0.31−0.93). However, FHA and only child were not significantly associated with food sensitization. Interestingly, there were a gender difference in food sensitization, with female children at lower risk than male children (OR = 0.54, 95% CI: 0.33−0.90).

### Changes in risk factors of food sensitization over 10 years

However, two independent binary logistic regression analyses could not identify the change in food sensitization factors over time. Therefore, additional analyses were performed for models 1 and 2 (2009 and 2019 surveys, respectively) to elucidate the changes in the risk factors for food sensitization from 2009 to 2019, as shown in **Table 3** (columns 6-8) and **Figure 2**.

The differences between the logistic regression coefficient *β*-values of each independent variable and the related statistical significance (*P*-value) were determined in model 3. The probability of food sensitization in the only-child group was significantly increased (*β*-value difference = 0.81, *P* = 0.024) during the 10 years, that is, the risk of food sensitization in the only-child group in 2019 increased by 226.0% (0.97/0.43 = 2.26) compared with that in 2009. Although the effect of only child on SPT results was not statistically significant in 2019, it should be noted that only child may become one of the risk factors influencing food sensitization over time.

Over the decade, the influence of sex and age on food sensitization also changed. The proportion of female children with positive SPT results decreased from 50% (36/72) in 2009 to 36.3% (29/80) in 2019 (**Table 2**). The probability of female children having a positive SPT result was higher in 2019 than in 2009 (0.54/1.56 = 0.35), and the change was statistically significant (*β*-value difference = 1.06, *P* = 0.008). The effect of age on food sensitization decreased (*β*-value difference = −0.69, *P* < 0.001), which was only 50.0% (1.36/2.70 = 0.50) in 2019 compared with that in 2009.

Although the effect of FHA on SPT results was different in independent analyses of the two surveys (models 1 and 2), that is, positive FHA increased the risk of positive SPT results in 2009 but had no effect on SPT results in 2019, no statistically significant difference was observed in model 3 (*β*-value difference = −0.57, *P* = 0.174). This means the effect of FHA on food sensitization did not change over the 10 years.

Other reported lifestyle factors, such as cesarean section, parental smoking, pet ownership, and vitamin D supplementation, etc., had no change in the influence of SPT results over the 10 years (**Table 3**).

## Discussion

### Principal findings

To the best of our knowledge, this is the first study to explore the changes in the risk factors of food sensitization based on a comparison of data from two cross-sectional surveys. Our results indicate that genetic and common lifestyle factors influencing food sensitization did not change over the 10 years, despite the differences in the risk factors in separate model. However, the effects of sex, age, and being the only child on food sensitization changed during this period.

The proportion of female children with positive SPT results decreased from 2009 to 2019, and this change was statistically significant (*P* = 0.008). Recent epidemiological studies have revealed that sex differences in the occurrence of food allergies. Kotz et al. reported that a lower prevalence of food allergy in female subjects than in male subjects during the period of 2001–2005 (15). A large study conducted in the Boston Integrated Health network showed that female patients had a higher prevalence of food allergy compared with male patients (4.2% and 2.9% in 2000 and 2013, respectively) but had a lower risk of food sensitization (OR = 0.32, 95% CI: 0.21–0.50, *P* < 0.01) (16). The sex differences in food allergy and food sensitization may be related to serum IgG4 concentrations, hormones, dietary habits, or differences in microbiome composition (17–19).

In addition, the role of sex differences in food-related anaphylaxis is likely to be age-specific, as male subjects are more affected by atopic disorders before puberty. Becklake et al. (17) reported that male children under the age of 10 had a higher risk of anaphylaxis incidence than female children; however, female children aged 10 years or older tended to have a comparable or even higher rate of anaphylaxis compared with male children. In our two models, age was associated with food sensitization (OR = 2.70 in 2009, OR = 1.36 in 2019); that is, older children are more likely to develop food sensitization. This may be attributed to the IgE levels in the body increasing with age (20). This result is in accordance with that reported in a European systematic review wherein increasing age was identified to be a potential risk factor for food allergies during 2000–2012 (12). Jerschow et al. (21) suggested that food-induced anaphylaxis was closely associated with older age and male gender during the period of 1999–2010. Nevertheless, the comprehensive analyses of the data of both surveys revealed that the effect of age on food sensitization gradually weakened during the 10-year period (*P* < 0.05), and the probability in 2019 was only 50.0% of that in 2009. Further studies are warranted to determine whether the effect of age on food sensitization will continue to change.

The incidence of food sensitization in the only-child group significantly changed during 2009-2019. This result supports the hygiene hypothesis and is consistent with those of previous studies, which have reported that children with an older sibling or more than one sibling had a decreased risk of food allergies (22, 23). Another possible explanation is that changes in demographic factors may influence individual microflora owing to immunological diseases; in multivariable analysis, the number of previous pregnancies was negatively associated with food sensitization (adjusted OR = 0.87, 95% CI: 0.76–1.00, *P* = 0.05) (24).

### Hereditary factors

FHA is a strong risk factor for the development of food allergy or food sensitization, wherein the heritability estimates for food allergy or food sensitization range from 15% to 82%, and the risk of developing food allergies when one has siblings with food allergies ranges from 1.3-fold to 12-fold (25). However, in 2013, Goldberg et al. (26) reported that parental atopic status alone does not predict the probability of children developing IgE-mediated allergy to milk. Although the change in the effect of FHA on food sensitization was not significant in the present study, it is worth noting that the effect of FHA on food sensitization was significant in the 2009 survey (model 1) but not in the 2019 survey (model 2), indicating alterations in the gene–environment interaction. This result is meaningful for future studies assessing lifestyle factors of atopic diseases.

### Dietary factors

In a recent multicenter, cluster-randomized trial, Skjerven et al. (27) found that early complementary feeding of cow’s milk from 3 months of age reduced the incidence of food allergy at the age of 3 years. Depending on specific genetic background, the time of food introduction may have varying impacts on the incidence of allergic diseases. We found that the introduction of formula before 4 months of age may protect against food sensitization as shown in the models 1 and 2 of the individual investigations, but its change between 2009 and 2019 did not reach statistical significance (model 3).

There is an ongoing controversy regarding whether breastfeeding is protective against atopic diseases. Sahara et al. (28) reported that ingestion of cow’s milk before 3 months of age prevents the development of allergy to cow’s milk in high-risk infants, and early exposure to formula as a supplement to exclusive breastfeeding has been reported to promote immunological tolerance (29, 30). The opposite view (that cow’s milk feeding in the first 3 days of life increases the risk of milk allergy) also has a scientific basis, as reported in a randomized clinical trial (31), but the duration of prolonged breastfeeding was shown to have no positive protective effect against food allergies (32). In the current study, we found that non-exclusive breastfeeding before 4 months of age did not increase the risk of food sensitization compared with exclusive breastfeeding in both models. Breastfeeding, undoubtedly, has various health benefits for children, but the relationship between breastfeeding and food sensitization could be better understood in the context of different individual genotypes (33). Further research can facilitate a better understanding of the genetic and environmental interactions, which may lead to the development of appropriate educational strategies and guidelines for treating children with varying risks of allergic diseases.

### Limitations

A limitation of our study includes our conclusion on the change in food sensitization risk factors is based on two cross-sectional surveys conducted 10 years apart; the temporal difference of the surveys may have influenced the trends of the risk factors. However, comparing cross-sectional data from the same clinic using the same diagnostic method can provide epidemiological data to describe the dynamic change in early-life factors. Another limitation is that the risk factors included in the two surveys were obtained from the literature or clinical experience and therefore may not fully reflect real-life conditions.

## Conclusions

In conclusion, the effects of genetic and common lifestyle factors on food sensitization remained constant between 2009 and 2019. However, the influence of demographic characteristics (sex, age, and being the only child) on food sensitization changed, which needs to be considered in future epidemiological and cohort studies.

## Data availability statement

The original contributions presented in the study are included in the article. Further inquiries can be directed to the corresponding author.

## Ethics statement

The studies involving human participants were reviewed and approved by the Medical Research Ethics Committee of our hospital and registered in the Chinese Clinical Trial Registry. Written informed consent to participate in this study was provided by the participants’ legal guardian/next of kin.

## Author contributions

ZM contributed to the conception and design of the study, carried out the data curation and formal analysis, drafted the initial manuscript, and reviewed and revised the manuscript. LC, RX, HF, JW, and JC designed the data collection instruments, collected the data, and reviewed and revised the manuscript. HL and YH conceptualized and designed the study, coordinated and supervised the data collection, and critically reviewed the manuscript for important intellectual content. All authors contributed to the article and approved the submitted version.
